# Notes and Comments

**Published:** 1898-06

**Authors:** 


### Notes and Comments.[1]

A NEW ARTICULATING PAPER.—The following is suggested by the *American Dental Weekly* to be used for articulating in default of the usual paper: Take a little thin paper, wet the finger with alcohol or water, and rub on a little polishing rouge. This dries quickly, and takes but a few minutes' time to prepare.

THE REMEDIES TO USE.—Dr. Welch, in a recent issue of the *Dental Brief*, says that it is much better to have a few good familiar remedies on hand than many that you are in doubt about, or that you are experimenting with. So with your treatment of a given class. Make a study of them till you are clearly satisfied with what you should do, and then do it. Continually varying your remedies and your practice is confusing, and tends to indecision and unreliable practice. Not that we should never take on anything new, or never discard anything old, or never vary our practice, but have some things settled, and as many things settled as

[1] The assistant editor solicits contributions for this department,—new methods, new remedies and formulas, or any short practical note which may prove of value to the practitioner or student. Address 1338 Walnut Street, Philadelphia.

possible, and then in investigating new things or processes go at it deliberately, systematically, and intelligently, and settle that. This never knowing and being forever coming to a knowledge of the truth is vexatious, bewildering, and childish.

---

Students practising Dentistry.—The *Dominion Dental Journal* says, "There should be a severe example made in the colleges of the first student caught practising for fee or reward on his own account. Such conduct in medicine or law is simply impossible, but in dentistry it has become a very general custom. Several cases have come under our own notice the last month where second-year students have made full upper sets for five dollars 'on the quiet,' and for parties who were quite well able to patronize regular licentiates. In fact, the custom has become such a fad that the rooms of some of these students in boarding-houses are unlicensed offices on a small scale, and a system of tooting for patients has even extended on the sly to the infirmary patients.

"It is generally this class of students who degrade the profession by cheap fees when they get into regular practice. It is a fact, too, that we have some very indigent young men in the ranks. Their poverty is no crime, but unfortunately they imagine it ought to justify them in breaches of law."

---

Suppressing Irregular Medical Practice, A Nice Legal Point.—The Medical Syndicate of the Southeast (France), wishing to convict a curate healer of unlawfully practising medicine, sent to the said curate two men, who on several occasions presented themselves at his consulting-rooms. They were examined by the curate, who subjected them to auscultation and percussion, and gave them a prescription, for which they paid him each time two francs. Fortified with this testimony, the Syndicate prosecuted the curate. The decision of the court was that the accused had instituted medical treatment in the case of the witnesses, but that as neither the one nor the other had really been sick, the curate could not be charged with illegal practice of medicine; in consequence "of one of the elements of the misdemeanor—namely, a disease—not being in evidence, the infringement with which the curate had been charged could not have been accomplished, for lack of an object." Upon this the curate, who was convicted of having practised medicine illegally, but in this particular case of having made use of it without practising it because the subject was not diseased, was acquitted.—*New York Medical Journal*, February 26, 1898, p. 290.

---

INVESTMENT, CLEANLY AND TIME-SAVING.—Woolly asbestos, well saturated with water, forms an investment that in many cases fully replaces the usual plaster and sand, with the advantage that it is more cleanly to handle, does not run into the cracks and crevices we desire to fill with solder, and there is no waiting for it to harden. The blow-pipe flame may be safely directed upon it immediately. The pieces to be united, held together with hard wax, may be embedded in it with the same facility as in plaster and sand. Without a moment's delay, the investment may be dried out and the wax burned off at the blow-pipe, instead of chipping it away, flux and solder applied, and the soldering completed in less time than is usually required for plaster and sand to harden. The investment does not crack, but with as little or even less mass than required of plaster and sand securely holds the parts together. Woolly asbestos is not expensive, and as it can be used over again repeatedly, the cost is trifling. With a little practice its use may with advantage be extended to many cases in which heretofore plaster has been considered essential.

---

TOOTH-WASHES AS THE CAUSE OF EROSION.—In a paper presented to the Odontological Society of Chicago Dr. A. W. Harlan says, "Most of the erosions that we have on teeth, if not begun by the use of these feeble, diluted acid lotions and washes, are carried along by the injudicious and too frequent use of tooth-brushes, tooth-rubbers, tooth-pastes, and powders containing ingredients that are not soluble in the fluids of the mouth or in water. Half of the ridges and grooves, and even disfigured faces of teeth that we see, are brought about through these means."

---

ARTICULATION AND OCCLUSION.—The correct dental uses of these words seem doubtful in the minds of many writers on dental subjects. In the 1896 report of the American Dental Association Committee on Nomenclature it is said that, "The meeting of the masticating surfaces of the upper and lower teeth is frequently spoken of as an articulation; it is more accurately described by the word occlusion." Dr. W. Storer How, in the *Dental Cosmos*, says, Would it not be more exact to use the term occlusion to describe the mere meeting of a lower tooth with an upper tooth, or of lower teeth with upper teeth, and when the antagonizing, opposing, or impacting teeth meet in their normal break-joint relations to each other, describe the result by the term articulation? The appliance aiding in the production of proper relations of occluding artificial teeth may therefore be correctly termed an articulator.

It must thus be obvious that simple contact of the occlusal surfaces of teeth in the mouth may or may not be articulating contact, but the process of proper mastication requires the *occlusive articulation* of the natural or artificial teeth, as the case may be.

These illustrative examples should serve to clearly discriminate the two words and clarify the current confusion regarding their related yet distinct significations.

---

Our Porcelain Teeth.—There are no dentists of experience and artistic tastes who have not realized the serious faults in the porcelain teeth of all the manufacturers of to-day, and yet, to use a familiar phrase, few of them do any "kicking." There is no one thing in the making of a set of teeth that gives us as much annoyance as the selection of teeth—simply a set of plain rubber teeth—at the dental depots. I oftentimes look over the large stocks at our depots and fail to find what is needed. Seeking first the size and shaped mould, then the color, and finally (and this is where the main trouble lies) to see what sort of bicuspids and molars have been combined with the fronts.

I presume most dentists think the fourteen teeth are made in one mould. The fronts are made in one mould and the bicuspids and molars in another. They are combined in sets by the girls who place them on cards, but under the direction of the superintendent. But, after all these years of experience, there is no judgment whatever used in making the combinations. Go to any stock of teeth and you will find complete verification of my statement. Here is a set of large front and small back teeth,—small fronts and large back teeth, and often the bicuspids longer than the cuspids. . . . —*Dr. Haskell in Dental Review.*

---

A Method of Crowning Broken-Down Teeth.—We are frequently called upon to crown badly broken-down teeth, or where devitalization of the pulp is not expedient, or is objected to by the patient, as in short lateral incisors, or denuded teeth. To meet such requirements the following method is recommended by Dr. C. J. Hand in the *Ohio Journal.* He says, in part, "After trimming, so that a band can be accurately fitted, make a band of thin platinum to cover all exposed portions of tooth or root, and solder with pure gold. (Of course, it is supposed that the labial wall has been bevelled as much as possible to permit the facing being brought in line with the adjoining teeth.) Instead of using a thin facing, secure a Bonwill crown, one that will nicely fit the space, and grind out the lingual portion, leaving the sides intact. Grind till the tooth can be placed over the platinum cap in proper position. It can now be waxed to the cap, invested, and body added to restore form ; or, better still, a little body mixed thin and placed on the porcelain and platinum cap, gradually raising the heat till fused. This method not only saves time, but prevents the investment absorbing the water in body and causing a porous bake. The crown is much better than a facing, as it prevents the difference in color at sides, which it is almost impossible to prevent when body is added to a facing. If care has been used the crown will go to place accurately and present a very natural appearance.

---

Fresh Air, Not Violent Exercise.—What a man of to-day needs most, says the *Annals of Hygiene*, is not athletics in a gymnasium, but plenty of fresh air in his lungs. Instead of a quantity of violent exercise that leaves him weak for several hours afterwards, he needs to learn to breathe right, stand right, and sit right. The young man or young woman who starts on a career of training and keeps it up year after year, just at the time when the body has a great deal of its own natural work to do and wants to do it, may make up his or her mind that beyond a showy and superficial development of muscle and strength, all this training is going to count against them in after life.

---

India-Rubber and Iniquity.—From the *Pharmaceutical Era* the following information concerning the gathering of our crude rubber is taken. A Danish missionary has made some revelations concerning the rubber trade on the upper Congo. He says the white man wants india-rubber, and is in a hurry to be rich ; and to terrify the black into rendering the utmost possible amount of labor the rubber-gatherers whose quantity falls below a certain weight are either shot or deprived of their hands. Rows of hands stuck on trees or heaps of them forwarded in baskets to European officers, or to native sergeants under their command, serve as an object-lesson to all. Rubber-gathering is a slow and difficult task, and whole villages are depopulated in order that their inhabitants, men, women, and children, may be sent on the search. Companies of black troops organized by white officers impress the villagers into this new species of slavery, and the reverend gentleman declares that he has seen forty-five villages burnt down and two abandoned through the rubber trouble. If these statements are reliable, the amount of iniquity represented by a stock of rubber goods must be alarming.

